# Polymer Solutions in Microflows: Tracking and Control over Size Distribution

**DOI:** 10.3390/polym17010028

**Published:** 2024-12-26

**Authors:** Artem Bezrukov, Yuriy Galyametdinov

**Affiliations:** Department of Physical and Colloid Chemistry, Kazan National Research Technological University, 420015 Kazan, Russia; yugal2002@mail.ru

**Keywords:** polymer solution, microfluidics, lab on chip, polyelectrolyte, liquid crystal, confinement, diffusion, convection–diffusion, modeling, size distribution

## Abstract

Microfluidics provides cutting-edge technological advancements for the in-channel manipulation and analysis of dissolved macromolecular species. The intrinsic potential of microfluidic devices to control key characteristics of polymer macromolecules such as their size distribution requires unleashing its full capacity. This work proposes a combined approach to analyzing the microscale behavior of polymer solutions and modifying their properties. We utilized the idea of modeling cross-channel diffusion in polydisperse polymer microflows using dynamic light scattering size distribution curves as the source data. The model was implemented into a Matlab script which predicts changes in polymer size distribution at microfluidic chip outputs. We verified the modeling predictions in experiments with a series of microchips by detecting the optical responses of injected nematic liquid crystals in the presence of microfluidic polymer species and analyzing the polymer size distribution after microfluidic processing. The results offer new approaches to tuning the size and dispersity of macromolecules in solution, developing auxiliary tools for such techniques as dynamic light scattering, and labs-on-chips for the combined diagnostics and processing of polymers.

## 1. Introduction

Microfluidics is a vibrant interdisciplinary area that offers a precise control over media with dissolved soft matter and macromolecular species [1,2,3,4] and attracts the continuing interest of researchers in a variety of fields, including but not limited to medicine, biology, and materials science, which is demonstrated in recent publications [5,6,7,8,9]. Microfluidic channels offer unique conditions, such as strongly laminar flows and predictable convection and diffusion behavior of dissolved particles, which cannot be achieved in “macroscopic” dynamic fluid systems [10,11,12]. These advantages of microfluidic devices make them highly demanded in soft matter engineering [4,13], biosensing [14,15,16,17,18], macromolecular chemistry [4,19,20], separation techniques [21,22], and the design of microfluidic devices powered with machine learning and artificial intelligence (AI) compatibility potential [23,24,25,26].

Combining microfluidics with polymer science is a promising approach to unleash the potential of polymers in soft matter and microtechnology [27,28,29]. In this respect, an important microfluidic concept is labs on chips [15,23]. Such devices can perform both physicochemical processing and analytical operations with macromolecular species [20,30,31,32,33,34] in a single portable chip. In particular, an important phenomenon of polymer diffusion can be analyzed in H-cell microfluidic devices [30], where the transverse diffusion of macromolecules from the laminar flow of a polymer solution into the contacting flow of a solvent can be studied. H-cell chips allow to apply relatively simple numerical modeling to predict the diffusion of dissolved species [21]. The application of such devices may provide cost-effective auxiliary tools to such polymer diffusion and size analysis techniques as dynamic light scattering [35,36], pulsed filed gradient NMR [37,38], or small-angle neutron scattering [39,40].

On the other hand, the analytical potential of microfluidic devices for polymer macromolecules is increasingly unleashed by microchips with integrated optically active materials such as liquid crystals (LCs) [41,42,43]. In microfluidic confinement, liquid crystals can change their molecular alignment in the presence of polymer macromolecules and polymer–surfactant systems [18,43,44,45,46]. Such changes generate a detectable optical response of LCs inside microchannels [15,16,42,47,48,49]. The analysis of their microscopy images allows to detect and quantify [50,51] the presence of molecular and macromolecular agents in microflows and offers a compatibility with prospective AI image processing tools [52,53].

Polymer systems are usually polydispersed and the distribution of macromolecules by size is among the key characteristics of polymer samples. In this respect, the diffusion of polydispersed macromolecules inside an H-cell microchip may change their size distribution at its outputs. Although H-cell microfluidics was one of the first areas that progressed from fundamental research to applied studies [54,55], it is mostly considered as a separation technique for different molecules. Therefore, a systematic study in modeling and testing polymer size distribution changes in H-cell microchips will allow the identification of the factors that control these changes. In turn, the H-cell + LC combination may provide a simple and cost-effective approach for the simultaneous processing and analysis of polymer microflows in such applications as the preparation of biopolymer samples for their biomedical testing and the fractionation of dissolved polymers.

This work continues a series of our previous research in polymers and liquid crystals and their behavior in microfluidic devices [56,57,58,59] and aims at developing a microfluidic approach for the combined detection of polymer macromolecules and modification of their size distribution. We developed a model for polymer diffusion in H-cell microfluidic chips based on their initial size distribution data. We successfully confirmed this approach for microfluidic chips designed and fabricated according to the numerical simulation results and performed their experimental testing by analyzing samples collected at chip outputs and detecting optical responses of the injected liquid crystal in the presence of polymer microflows.

## 2. Materials and Methods

### 2.1. Materials

Polyacrylic acid (PAA) was purchased from Polysciences, Inc. (Warrington, PA, USA). PAA’s molecular mass is 90,000 g/mol. The polymer was sold as a viscous liquid (25% aqueous solution) and used as received. We selected this material as a model substance because its macromolecular properties and behavior in solutions are well-characterized [60]. This polymer is thus convenient for numerical simulations and is expected to ensure a smooth comparison of experimental and numerical data.

Cetyltrimethylammonium bromide (CTAB) was purchased from BDH Limited, Poole, England as a powder and used as received.

For the liquid crystal phase, the nematic liquid crystal N-(4-methoxybenzylidene)-4-butylaniline (MBBA) was used. It was purchased from Reachem, Moscow, Russia and used as received. It exhibits liquid crystal properties at the standard 25 °C temperature, at which all the polarizing microscopy observations were performed.

Polydimethylsiloxane (PDMS) Sylgard 184 was purchased from Dow Corning (Midland, MI, USA) and used to fabricate microfluidic devices. It comes as a two-part elastomer kit (the pre-polymer and curing agent). SU-8 3050 photoresist (Microchem Corp., Westborough, MA, USA) was used to produce a mold for microfluidic chips.

### 2.2. Solutions

For microfluidic experiments, bulk samples of 1 g/L PAA were produced from their initial solutions and allowed to dissolve overnight, where 50 mmol/L KBr was used as a background electrolyte. A CTAB solution with the concentration of 0.014 mol/L was used for liquid crystal orientation experiments and forming complexes with the PAA. This concentration was selected to be equal to the concentration of PAA monomer groups in its 1 g/L solution. Bidistilled water was used for all solutions. Before preparing solutions, water was filtered by 0.2 µm Millipore PTFE filters.

PAA solutions and a solvent were infused into microfluidic devices by Shenchen ISPLab01 syringe pumps (Baoding Shenchen Precision Pump Co. Ltd., Baoding City, China), which provide a minimal flow rate of 0.001 µL/min. The flowrates of the polymer and surfactant solutions and a solvent varied in the range of 0.5–6 µL/min.

To collect solutions downstream of the H-cell microfluidic devices, we connected PTFE tubes of identical lengths (10 cm) and internal diameters that fit the needle tips inserted into microchip outputs (20 G type needles, 0.9 mm diameter). Identical tubes provide a uniform release of solutions from multiple outputs. The collecting reservoirs were disposable polystyrene Malvern Zetasizer micro cuvettes with an analytical volume of 40 μL. Such cuvettes allowed to collect a sufficient volume of samples for the dynamic light scattering (DLS) analysis within microfluidic experiment times of up to 30–60 min. All the plastic parts were used new and washed with filtered water before experiments with polymer solutions.

### 2.3. Methods

The hydrodynamic diameters of the PAA were measured using a Malvern Zetasizer Nano ZS light scattering system, Malvern Panalytical Ltd., Malvern, UK. The reported diameters of particles measured by DLS correspond to the maximums of size distribution by intensity. For DLS characterization, microfluidic samples were collected at microchip outputs with Malvern microcuvettes, which were convenient for sampling solutions at low flowrates within the range of microliters per minute. The samples were analyzed by DLS after setting up microflows of the polymer solution and pure solvent until the DLS reports showed a reproducible size distribution. After that, dynamic light scattering measurements were performed at least three times to obtain reproducible results.

Microchannel images taken during the processing of the polymer solutions were recorded on a Levenhuk D320 optical microscope (Levenhuk Inc., Tampa, FL, USA). The microchannels were imaged at 10× magnification using a Levenhuk M1400 Plus camera (Levenhuk Inc., Tampa, FL, USA) with a resolution of 0.27 µm/pixel.

The convection–diffusion equations for macromolecular microflows were solved using Matlab 2021a software with the Partial Differential Equations Toolbox.

The orientation behaviors of the liquid crystal media in microfluidic flows were studied by polarized optical microscopy using an Olympus BX51 microscope (Olympus, Tokyo, Japan) equipped with a high-precision Linkam heating system that allows to provide uniform temperature conditions for experiments. Microscopy images were captured at 100× magnification using a ToupCam E3ISPM08300KPC camera (Touptek, Hangzhou, China).

### 2.4. Device Fabrication

Microfluidic devices were fabricated using standard photolithography techniques [61,62]. We designed and fabricated microfluidic devices with maximum main channel lengths of 300 mm, which could be comfortably accommodated in a compact microfluidic device (microscope glass size) as serpentine channels. SU-8 photoresist and a transparency photomask with the negative image of a microchip were used to produce a 100 µm thick mold of microfluidic chips on top of a 3-inch silicon wafer. Sylgard 184 PDMS pre-polymer was mixed with a curing agent, poured over the mold, and allowed to cure for 4 h in a 60 °C oven. Once cured, the PDMS was peeled off the mold and bonded to a flat PDMS slab via plasma treatment. The PDMS device was then heated in an oven at 180 °C for 1 h to finalize the bonding of the two polymer layers. The widths of all the channels in the microchips were 200 µm. Filtered bidistilled water was infused into freshly produced microchips for 5 min to wash the microchannels before infusing the polymer solutions.

## 3. Results

### 3.1. Modeling and Designing H-Cells for Polymer Diffusion Studies

**Figure 1:**
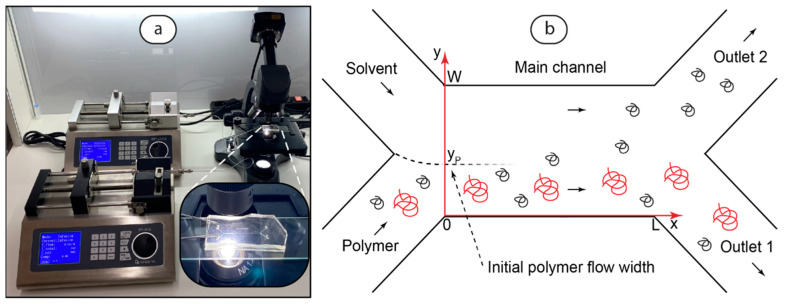
Microfluidic setup (**a**) and the chip geometry with transverse diffusion of polymer macromolecules into the solvent flow (**b**). S is the solvent; P is the initial polymer solution; Outlet 1 and Outlet 2 are the solutions sampled at the chip outlets; x and y are the coordinate axes; L and W are the main channel length and width, respectively; and y_P_ is the initial polymer solution flow width. Red arrows indicate coordinate axes. Red and black particles represent macromolecules of different sizes.

At the first stage of this work, we focused on identifying the parameters of microfluidic devices suitable for studying polymer diffusion and modifying the size distribution of macromolecules. We started with a basic H-cell model with a straight main channel and continued with more complex microchip designs. The microfluidic setup that was used for the experiments and the H-cell geometry are shown in Figure 1.

The microfluidics set-up shown in Figure 1a includes two syringe pumps for infusing the polymer solution and the solvent at different flowrates and performing a precise control of the flowrates. The initial polymer solution was infused into the lower input channel and the solvent was infused into the upper input channel (Figure 1b). The polymer macromolecules diffused in the solvent flow. The sample was collected at Outlet 2 and further analyzed by DLS. The optical microscope equipped with the digital camera was used to control the experiments. Finally, the microfluidic chips shown in Figure 1a represent H-cells with different channel lengths and designs.

In the initial polymer solution, PAA macromolecules were represented by nanoparticles with a 31 ± 9 nm peak at the size distribution by intensity according to a series of DLS experiments. PAA is reported to be a weakly charged polymer [60] with compact macromolecular coils in the presence of a background electrolyte. It should be noted that we used solutions of PAA with a background electrolyte (50 mmol/L KBr). With such additives of KBr, PAA was found to be represented by macromolecular coils which were detected by DLS with robustness and good reproducibility of results. The size distribution by DLS was found to be reproducible in a series of experiments in the broad range of PAA concentrations of 0.05–5 mg/mL. In this concentration range, polymer macromolecules did not aggregate (no larger size peaks appeared in the DLS scattering plots). Evaluation of the number of polymer macromolecules in a 1 mg/mL solution according to [33] demonstrated that polymer particles occupy less than 2% of the solution volume. Such solutions are considered to be dilute with non-interpenetrating polymer chains. In this work, therefore, we approximate the diffusion model, assuming that polymer macromolecules diffuse independently from each other and avoid the complications required for more concentrated microfluidic polymer systems [63,64,65].

To characterize the diffusion of polymer particles in a microchannel, we used a model with parallel laminar flows of the polymer solution and the respective pure solvent [21,66,67]. The size distribution of the polymer in the initial solution was used in numerical simulations.

For the stationary conditions of microfluidic flow and a constant diffusion coefficient, D, the concentration field of dissolved particles can be described by the following convection–diffusion equation:

(1) $$U\frac{\partial C_{P}}{\partial x}=D_{P}\frac{\partial^{2}C_{P}}{\partial x^{2}}+D_{P}\frac{\partial^{2}C_{P}}{\partial y^{2}},$$

where C_P_ is the concentration of polymer and D_P_ is its average diffusion coefficient.

Equation (1) was further modified by neglecting the axial diffusion term. Such a modification was reported in [21]. In particular, this work summarizes microfluidic approaches to the diffusion of dissolved media and, in turn, refers to publications focusing on the theoretical analysis of pressure-driven laminar flows in microfluidic devices, such as [67]. The reasoning for neglecting the axial diffusion term is the steady-state input of the polymer solution and solvent into the microchip, which then form predominantly parallel uniform laminar flows after entering the main channel. This approach was successfully used in our previous work [56]. In the further set of Equation (2), which describes the concentration field of a polymer sample in a microchannel, we considered the radial diffusion (in the y-direction) and neglected the axial diffusion (in the x-direction).

For a polydispersed polymer sample, a polymer concentration field can be described by the following system of convection–diffusion equations which neglects the axial diffusion:

(2) $$\left\{\begin{array}{c} U\frac{\partial C_{P_{1}}}{\partial x}=D_{P_{1}}\frac{\partial^{2}C_{P_{1}}}{\partial y^{2}}\\ U\frac{\partial C_{P_{2}}}{\partial x}=D_{P_{2}}\frac{\partial^{2}C_{P_{2}}}{\partial y^{2}}\\ \ldots \\ U\frac{\partial C_{P_{i}}}{\partial x}=D_{P_{i}}\frac{\partial^{2}C_{P_{i}}}{\partial y^{2}} \end{array}\right.,$$

where U(y) is the flow velocity, $C_{P_{i}}$ is the molar concentration of the PAA monomer groups in a fraction of a polydisperse sample, $D_{P_{i}}$ is the diffusion coefficient of PAA macromolecules in this fraction, according to the DLS data, and x and y are the coordinates.

The flow velocity U distribution in a microchannel can be approximated by the following equation [21]:

(3) $$U(y)=\frac{3}{2}[1-{\left(2\frac{y}{W}-1\right)}^{2}U]$$

where U = Q/(WH) is the flow velocity calculated from the flowrate and H is the height of the microchannel.

In this work, we assume that no-slip boundary conditions U(0) = U(W) = 0 are applied.

The boundary conditions for the junction of the flows were introduced according to the microchannel geometry shown in Figure 1:

(4) $$C_{P_{i}}\left(x=0,y\right)=\left\{\begin{array}{c} C_{P_{i}}^{0},y\geq y_{P}\\ C_{P_{i}}^{0},y<y_{P} \end{array}\right.,$$

where y_P_ is the transverse coordinate of the polymer flow boundary that is calculated from the flowrate ratio of polymer solution and the aqueous phase [21]:

(5) $$y_{P}=W\frac{Q_{P}}{{Q_{P}+Q}_{S}},$$

The boundary conditions for the microchannel walls are introduced assuming zero flow of dissolved species through the walls [21]:

(6) $${\frac{\partial C_{P_{i}}}{\partial y}}_{\left(0,W\right)}=0,$$

The system of Equation (2) with boundary conditions (4) and (6) was solved numerically in Matlab. Details of the mathematical model and the respective Matlab script for calculating the polymer size distribution and dimensionless parameters of microchip operation are provided in the Supplementary Materials.

In this work, we performed a numerical analysis of polymer diffusion in the total flowrate range of 1–20 µL/min. Such a range of flowrates is convenient for the smooth infusion of liquids to microchips by microfluidic syringe pumps and also provides a relatively fast sampling of solutions at H-cell outputs for further DLS studies. For a reliable and reproducible DLS analysis and further potential applications of such H-cells, a substantial amount of polymer should diffuse into the solvent flow (Figure 1b). With the model (Equations (2)–(6)), we evaluated conditions at which the concentration of polymer diffused to Outlet 2 (Figure 1b) will be about 10% of the initial one in the flowrate range of 1–20 µL/min. The results are summarized in Table 1.

For the chip design shown in Table 1, laminar flow in the x-direction is assumed. We agree that the laminar flow in the main channel (Figure 1) may still be aligning to the x-direction around x = 0 (the merge of inlets flows) and starts to deviate from the x-direction at x = L (division of the main channel flow to the outlet flows). Therefore, Equation (1) is valid for well-established laminar flow in the x-direction. This simplification, however, can be appropriate considering the total length of the main channel with respect to its width (L/W = 50–1000) in the microchip used in this work. Thus, the flow in the main channel is predominantly laminar in the x-direction.

The 10% diffusion condition is fulfilled with the 15 mm-long main channel for the lower flowrate boundary of 1 µL/min and with the 300 mm-long main channel for the upper flowrate boundary of 20 µL/min. To make a sequence of lengths for further experimental and numerical studies, we added 100 mm and 30 mm channel lengths with the respective flowrates between these boundaries. To accommodate 30–300 mm main channels in a sufficiently small chip, we needed a serpentine design with curved main channels.

The Reynolds numbers calculated for the Q_0.1_ flowrates in Table 1 show that all the flows are strongly laminar (Re ≤ 1).

To verify that the flows remain laminar in serpentine microchannels and no vortices appear at the bends, we calculated Dean numbers, Dn, that characterize the behavior of flows in curved geometries. In all the flow conditions, Dn < 1, so no vortex formation is supposed to occur, the flows remain laminar in serpentine channels, and the model in Figure 1b can be applied to all the channel lengths.

Peclet numbers that characterize the ratio of convention and diffusion rates were found to be Pe >> 1. Therefore, no complete diffusion of polymer occurs into the solvent flow. Under such conditions, different diffusion rates of polydisperse macromolecules are supposed to provide a size distribution at Outlet 2 different from the original one.

The dimensionless numbers shown in Table 1 can be used to create parallel flow conditions in a variety of possible channel geometries in microchips to verify if strong laminar flow conditions are fulfilled.

**Figure 2:**
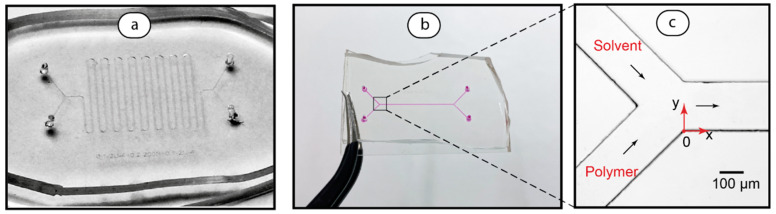
Designs of serpentine (**a**) and straight (**b**) microfluidic chips according to mathematical simulations’ results and the microscopy images of their inlet junctions (**c**). Straight-channel chip is filled with dye for a better visibility.

The microfluidic chips with channel geometries shown in Table 1 were designed and fabricated. Examples of serpentine and straight channel chips are shown in Figure 2.

Both the serpentine (Figure 2a) and straight-channel (Figure 2b) chips have the same H-cell design. The polymer solution and solvent are infused into the lower and upper input channels, respectively (Figure 2c).

At the next stage of this work, we used chips shown in Figure 2 for modeling and experimental testing of polymer size distribution modification.

### 3.2. Modifying Polymer Size Distribution in H-Cell Microchips

**Figure 3:**
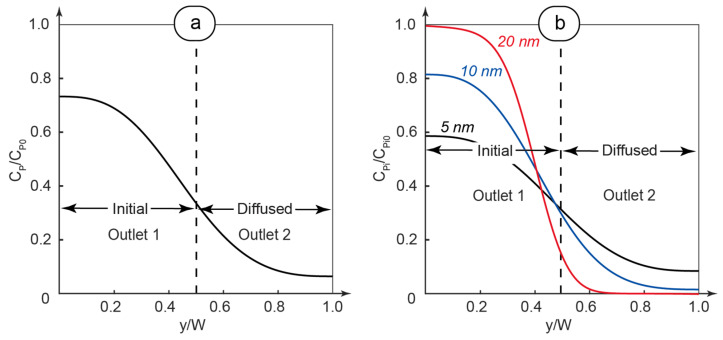
Dimensionless PAA concentration curves at the main channel cross-section for x = L: (**a**) total concentration across the main channel; (**b**) concentrations of selected fractions across the main channel. The main channel length is 100 mm. The ratio of polymer to solvent flowrates is 0.5 and the total flowrate is 2 µL/min.

To evaluate size distribution of PAA after its diffusion into the solvent flow, we analyzed the concentration curves of polymer fractions at the cross-section of the main channel end with the axial coordinate x = L, where the main flow splits into two outlet flows (as shown in Figure 1). The results of simulations are shown in Figure 3.

Figure 3a demonstrates the summarized dimensionless concentration of PAA across the main channel. This concentration was calculated as the sum of the numerical solutions of Equation (1) that were solved for each polymer fraction with a specific diffusion coefficient. It agrees well with the literature that discusses concentration curves in convection–diffusion conditions [67,68].

Figure 3b demonstrates the concentration distribution curves of selected different polymer fractions with specific diffusion coefficients of macromolecules. We should clarify that the “Initial” part of the flow, which predominantly contained the original polymer solutions, was released through Outlet 1 in the microfluidic experiments (Figure 1). The solvent flow with macromolecules diffused in it was released through Outlet 2 (marked as “Diffused” in Figure 3).

In Figure 3b, we can see that the concentration distribution of polymer macromolecules depends on their size and the resulting diffusivity. Smaller particles diffuse faster into the buffer flow and their resulting concentration is higher than the concentration of larger macromolecules, which diffuse slower. The resulting size distribution curve of polymer particles is supposed to shift to a smaller size area.

**Figure 4:**
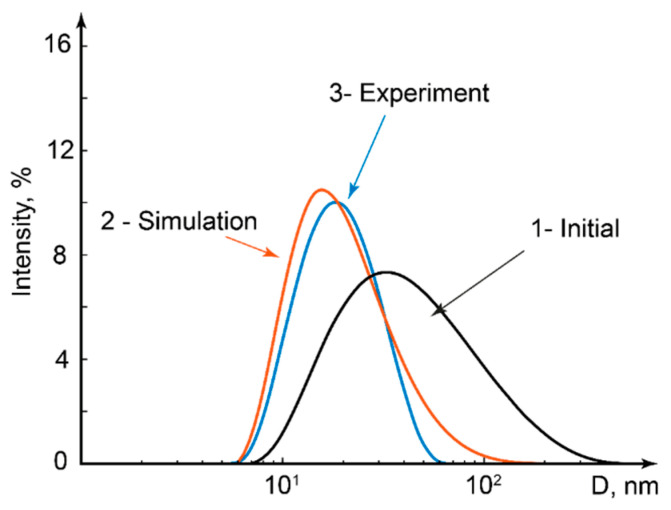
Example of PAA size distribution curves before and after microfluidic processing: 1—initial polymer solution according to DLS; 2—numerical simulation of the “diffused” flow after microfluidic processing; and 3—experimental verification of simulation results. The main channel length is 100 mm. The ratio of polymer to solvent flowrates is 0.5 and the total flowrate is 2 µL/min.

To evaluate the size distribution of polymer macromolecules in the “Diffused” flow, we calculated concentrations of each fraction in this flow by solving Equation (2) for each PAA fraction and then calculating the concentrations of PAA species in each fraction. The concentration curves shown in Figure 4 were transformed into the resulting size distribution, which is demonstrated in Figure 4. It should be noted that for numerical simulations and plotting concentration curves we used the size distribution by number. The results were transformed into the size distribution by intensity, which is a primary DLS output. Details are provided in the Supplementary Materials.

Figure 4 represents a comparative analysis of both DLS measurements and numerical simulations. The original size distribution of the PAA is represented by curve 1, which was plotted according to the DLS experiments. Curve 2 shows the numerical modeling result for the size distribution in the “Diffused” flow. Finally, curve 3 demonstrates experimental verification of the modeling results.

The size distribution of the original polymer is broad and the original polymer solution contains a certain percentage of particles that are much larger than the size corresponding to the intensity peak. On the other hand, such a broad size distribution can be advantageous for this work. According to the size distribution of processed samples, they contained much less of the larger fractions. Therefore, we can demonstrate that this microfluidic approach can be used for “cutting-off” large fractions of polymer macromolecules or possible large-size inclusions.

We can see a shift of the size distribution peak to a smaller size area after microfluidic processing. Curves 2 and 3 are narrower than curve 1, which indicates a lower dispersity of polymer macromolecules as compared with the original solution. There is a satisfactory agreement between the numerical modeling prediction and its experimental verification.

It should be noted that the behavior of polymer flows in capillaries can be more complex than that represented by Equation (2). In [69], the authors modelled the behavior of DNA macromolecules in micrometer-scale channels and discussed the migration of macromolecules towards the channel center. In that work, both the size scale of polymer particles and the microchannel width and height were comparable (micron-scale) and the respective more complex flow distribution effects were reported. In our work, the average size of the PAA macromolecules (tens of nanometers) is several orders of magnitude lower than the width of the microchannels (200 µm). We expect, therefore that the contribution of such an effect at the scale of entire channel width may be negligible.

In [70], the authors reviewed the migration of macromolecules under flow. The reported factors and effects that could be responsible for such a migration were the stretching of polymer macromolecules along the flow, non-uniform velocity gradient fields, or anisotropic diffusivity of macromolecules. The authors reported that notable effects of polymer migration could appear with relatively high molecular weight macromolecules (>10^6^), macromolecules of non-spherical shapes, such as rod-like molecules, or channels with a high ratio of length to width (of about several thousand). The authors also analyzed the dimensionless parameter R/B, which characterizes the ratio of characteristic size of macromolecules to characteristic width of flow geometry, and reported that the migration effect will be of very small magnitude for R/B << 1.

In our work, we used PAA with a molecular weight of 90,000 with the sizes of macromolecules much smaller than the microchannel width and height (R/B << 1) and polymer migration effects were expected to be negligible. The outcomes of these publications will, however, be important for future studies and the adaptation of the model represented in this work to more complex conditions, which were discussed in these papers.

Figure 4 demonstrates the results for the selected flow conditions and chip design. To identify and summarize the factors that govern the size distribution of polymer macromolecules, we developed a criterial equation for the channel model shown in Figure 1b. Let us analyze the conditions at which polymer macromolecules diffuse to the opposite wall point at Outlet 2 (x = L and y = W). The time t_dif_ required for such diffusion and the required microchannel length for diffusion to occur is [21]

(7) $$t_{dif}=\frac{{\left(W-y_{P}\right)}^{2}}{4D_{P}}\approx \frac{L}{3/2U}$$

where W is the microchannel width, y_P_ is the boundary between the polymer and solvent flows at the junction of the inlets, L is the microchannel length, and U is the flow velocity in the main channel.

By introducing a set of dimensionless numbers, we can transform Equation (7) into the scaling law for the diffusion of polymer macromolecules to the x = L and y = W point. These numbers are the Peclet number Pe = UW/D_P_, where D_P_ is the diffusion coefficient of polymer macromolecules, the microchannel length-to-width ratio L_N_ = L/W, and the ratio of the polymer and solvent flowrates is Q_N_ = Q_P_/Q_S_. Considering that y_P_ = WQ_P_/(Q_P_ + Q_S_), the resulting scaling law is

(8) $$Pe_{dif}\approx \frac{8}{3}L_{N}{\left(1+Q_{N}\right)}^{2}$$

From this law, we can determine factors that are favorable and unfavorable for diffusion to x = L and y = W. The increase of the channel length L and the flowrate ratio Q_N_ will increase the polymer concentration at x = L and y = W. In turn, an increase of the flow velocity U and channel width W will decrease the polymer concentration at this point. A combination of these interrelated factors can be used to control concentrations of polymer fractions at Outlet 2 and, therefore, the resulting size distribution of the polymer.

Derivation of the scaling law (Equation (8)) is described in more detail in the Supplementary Materials.

In all the experiments, we used microchips with the same channel width W to avoid flow effects related to the microchannel aspect ratio H/W [21], where H is the height of a microchannel. We varied, therefore, the channel length, the total flowrate, which is proportional to the flow velocity, and the polymer/solvent flowrate ratio. The results of selected simulations and modeling experiments are summarized in Table 2.

Experiment 1, demonstrated in Table 2, represents classical conditions of H-cell operation: equal flows of the PAA solution and the solvent and a relatively large concentration of diffused polymer at Outlet 2. According to numerical simulations and experiments, such conditions did not provide a substantial shift of the DLS intensity peak after microfluidic processing of the polymer solution with all the studied chip designs. A possible reason is that even small amounts of larger fractions which penetrate into the Outlet 2 flow make a predominant contribution to the size distribution by intensity.

In Experiments 2–5, we used a narrower flow of polymer solution (Q_N_ < 1) to “cut-off” larger size polymer fractions from Outlet 2. In Experiments 2–5, it was possible to shift the intensity peak from ~25 nm to ~10 nm, depending on the conditions and chip designs.

Table 2 also demonstrates that we can achieve similar size distribution results with different flow conditions (Experiments 3 and 4) or chip designs (Experiments 2 and 6) according to Equation (8).

Chips with higher lengths are also more suitable to achieve higher concentrations of polymer at Outlet 2 at the same flowrates.

Finally, the Experiment 6 conditions represented the lower threshold for DLS analysis of the Outlet 2 solutions. For the 15 mm chip, DLS provided no reliable results for higher flowrates or lower Q_N_, which can be attributed to the fact that the polymer concentrations were too low for DLS detection under such conditions.

Numerical simulations and experiments showed a satisfactory agreement for all the Experiments 1–6.

Therefore, Experiments 1–6, listed in Table 2, demonstrated shifts of the size distribution peaks for different chip designs and flow conditions. A desired size distribution change can be achieved by varying flow conditions in chips of various designs according to Equation (8).

At the next stage of this work, we proceeded to visualize polymer flows in microchannels, providing the studied microfluidic chips with both polymer processing and detection capabilities.

### 3.3. Microfluidic Detection of Polymer Macromolecules with Liquid Crystals

Microfluidic lab-on-chip instruments are capable of performing various physicochemical processing and analysis operations. In this work, the microfluidic control of polymer size distribution was verified by further out-of-chip analysis by DLS. Optical microscopy images of microflows (Figure 2c), however, did not provide a direct visualization of polymer flows, which can be useful to detect the presence of polymer macromolecules and control their flow in a microchannel.

To provide microfluidic H-cells with polymer flow tracking capabilities, we applied the approach that was described in our previous work [58]. The basic idea behind this approach is to analyze the response of optically active soft matter such as liquid crystals in the presence of polymer flows in a microchannel.

A predominant contribution to the orientation of liquid crystal molecules in microchannels is made by their walls [47]. Wall effects are of key importance in microfluidics [21] and surfactants are commonly used to control surface phenomena in microchannels by forming adsorption layers at their walls. In their turn, the geometry of surfactant molecules such as CTAB can be similar to rod-like molecules of nematic liquid crystals such as MBBA. Microfluidic confinement is, therefore, a convenient environment for tuning the alignment of liquid crystal molecules by introducing orienting agents, such as surfactants, that are capable of adsorbing at microchannel walls [47].

**Figure 5:**
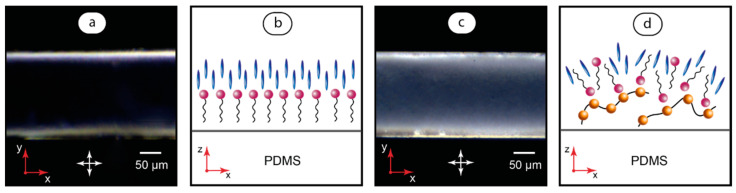
The optical response of MBBA liquid crystals to CTAB surfactant in the microchannel (**a**) and proposed alignment of liquid crystal molecules (**b**); the optical response of MBBA liquid crystals to CTAB + PAA in the microchannel (**c**) and proposed molecular alignment (**d**). Arrows indicate positions of crossed polarizers.

To test the orienting impact of CTAB and PAA on MBBA liquid crystals in microfluidic confinement, we infused their solutions into a microchannel and then replaced them with MBBA. Figure 5 demonstrates the respective light transmittance and assumed orientation of LC molecules.

Transmittance of light through a layer of liquid crystal between crossed polarizers is determined by Malus’ law [71]. For the positioning of crossed polarizers according to Figure 5, the transmitted light intensity follows the relation I ≈ I_0_sin^2^2θ, where I_0_ is the intensity of incident light and θ is the angle between the long axis of LC molecules. Changes in the orientation of LC molecules in a microchannel will change the angle θ and the resulting light transmittance.

In the first experiment, we filled a microchannel with CTAB and then replaced it with MBBA. The resulting birefringence pattern (Figure 5a) shows a uniform dark area in the central part of a microchannel which transforms into bright stripes near its sides. Such a pattern can be attributed to an orientation of LC molecules that is close to homeotropic [47]. In this case, LC molecules tend to align perpendicular to microchannel walls due to anchoring by surfactant molecules (Figure 5b) and the resulting light transmittance approaches zero. Bright stripes represent transition areas with the anchoring impact of both the vertical and horizontal walls of the microchannel where the mean value of θ varies, as does light transmittance.

In the next experiment (Figure 5c,d), we infused a PAA solution into the entire microchannel and then substituted it with a CTAB solution and finally replaced with MBBA. The increase of light transmittance shown in Figure 5c as compared with Figure 5a indicates changes in values of θ and, therefore, a different orientation of LC molecules in the microchannels processed with the CTAB solution and the CTAB + PAA pair of solutions, respectively.

This effect can be attributed to interactions that may occur between CTAB molecules and PAA macromolecules at microchannel walls (Figure 5d). The binding of CTAB molecules by PAA macromolecules is supposed to make their orientation more randomized with respect to microchannel walls as compared with the orientation of an individual surfactant (Figure 5b) and, in turn, randomize the anchoring angles of LC molecules. The resulting change in the mean value of θ influences light transmittance and is supposed to provide the birefringence pattern shown in Figure 5c.

It should be noted that the processing of microchannels with PAA solution only did not result in notable changes of the MBBA optical response. Therefore, PAA may possess poor orienting capabilities towards MBBA in a microchannel. Thus, in the case shown in Figure 5, the surfactant provides the polymer with an ability to influence the optical response of liquid crystals in a microchannel. In other words, CTAB plays the role of a mediator for the MBBA and PAA by interacting with both of them.

We performed an experimental study of the size distribution of PAA-CTAB complexes. For the experiments, we selected the concentration ratio of PAA monomer groups to the concentration of CTAB C_CTAB_/C_PAA_ = 1 according to our previous work [56]. At this concentration ratio, PAA and CTAB formed soluble aggregated complexes, while at lower concentration ratios they formed insoluble complexes which further aggregated and precipitated and could be easily distinguished both in bulk conditions and inside a microchannel. The typical size (the peak of the size distribution by intensity) of complexes at C_CTAB_/C_PAA_ = 1 was found to be 240 ± 18 nm in a series of microfluidic experiments. This value agreed with the typical sizes of these complexes synthesized in standard “macroscopic” conditions by mixing polymer and surfactant solutions. No considerable changes in the sizes of complexes were found for C_CTAB_/C_PAA_ = 1–2. Typical sizes of PAA-CTAB complexes were found to be much larger than the typical sizes of PAA macromolecules shown in Figure 4.

**Figure 6:**
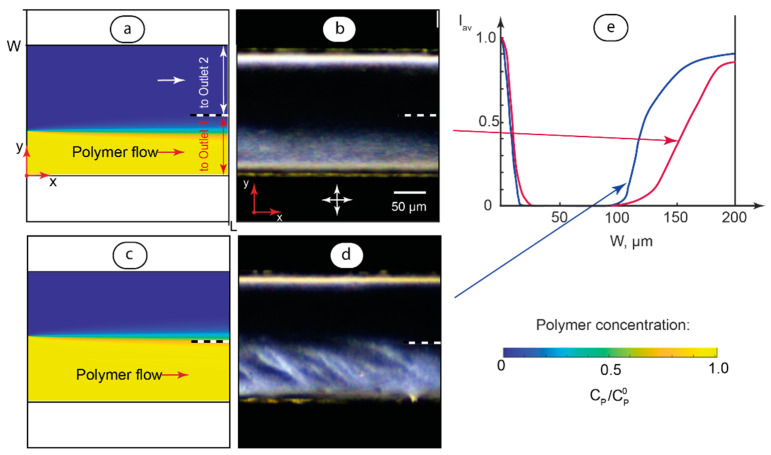
Optical response of MBBA liquid crystal molecules infused into the microchannel after PAA diffusion processing and subsequent filling of the microchannel with CTAB surfactant solution: numerical modeling of polymer flow and diffusion for polymer/solvent flowrate ratio = 0.5 (**a**) and the resulting MBBA response (**b**); numerical modeling of polymer flow and diffusion for polymer/solvent flowrate ratio = 1 (**c**) and the resulting MBBA response (**d**); and average normalized transmission light intensities in microchannels (**e**). The main channel length is 15 mm. The total flowrate is 6 µL/min. Images were captured in the middle of the main channel (x = 0.5 L). Arrows indicate positions of crossed polarizers. Dashed line indicates further separation of the flows to Outlets 1 and 2.

Careful substitution of the PAA + solvent pair in the microchannel with a CTAB solution was intended to provide excess CTAB in a microchannel C_CTAB_/C_PAA_ ≥ 1 with respect to PAA, which is supposed to adsorb at the microchannel walls. In Figure 6, the resulting microchannel environment shows no traces of precipitates that may emerge at lower C_CTAB_/C_PAA_ ratios. The optical response of MBBA that was further infused into microchannels (Figure 5c and Figure 6) can be attributed to the formation of such complexes.

This approach to analyzing the impact of PAA on the optical properties of MBBA liquid crystals was further used for H-cells that performed microfluidic processing of polymer. After the processing of polymer solutions, a CTAB solution was infused into the microchannel and replaced with the liquid crystals. Figure 6 compares numerical simulations of PAA flows and optical responses of the infused liquid crystals.

In Figure 6, numerical simulations were performed for various ratios of polymer and solvent flowrates (Figure 6a,c). We can see a good agreement between numerical simulations of polymer flows and the optical responses of MBBA infused into the microchannel (Figure 6b,d).

The polarized microscopy images in Figure 6c,d were processed in Matlab to analyze the light transmission intensity of microchannels with infused liquid crystals according to the methodology developed in our previous work [72]. According to this methodology, the dimmest and brightest microfluidic image spots are taken as references and the normalized average brightness of microchannel POM images is calculated with respect to these references:

(9) $$I_{av}=\frac{\bar{Y}-{\bar{Y}}_{min}}{{\bar{Y}}_{max}-{\bar{Y}}_{min}}$$

where $\bar{Y}$, ${\bar{Y}}_{min}$, and ${\bar{Y}}_{max}$ represent average brightness of the current, dimmest, and brightest image spots, respectively. As the brightness of image spots is proportional to the transmitted light intensity, we get normalized transmitted light intensities across the microchannel in the [0, 1] range (Figure 6e).

In Figure 6e, an increase in transmission intensities at the left part of the plot can be attributed to microchannel wall effects and surfactant adsorption at the vertical walls of the microchannel [47]. The right segments of the plots show different dependences of the transmitted light intensities of the channel width and agree with the simulation and experiment data in Figure 6a–d.

The optical responses of microchannels in Figure 6 demonstrate, therefore, a hybrid alignment of LC molecules. In the microchannel sections that were previously occupied by solvent flows, the light transmission pattern is similar to that shown in Figure 5a. In turn, in the microchannel sections that were previously occupied by polymer flows, the light transmission pattern is similar to that shown in Figure 5c and can be attributed to the presence of polymer macromolecules at the microchannel walls in these sections.

The approach shown in Figure 5 and Figure 6 was, therefore, used to correlate the optical response of MBBA in specific sections of the main channel with flows of polymer solutions through this channel and quantify light transmittance using applicable software. Using MBBA liquid crystals as an optically active component, we can, therefore, track polymer solutions in contacting polymer–solvent microflows.

## 4. Discussion

In this work, we offered a combined microfluidic approach to analyzing the microscale behavior of polymer solutions and controlling their size distribution properties. In this context, this work offers a new application scope of H-cell microchips by shifting the focus from separating different types of molecules to a predictable control of size distribution of polydispersed samples. The integration of liquid crystals into the microchannel environment provides these microchips with additional sensing capabilities that allow to detect polymer macromolecules at specific sections of microchannels. Such a combination of sensing and processing can be useful for on-chip tracking and controlling polymer modification processes with a lab-on-chip represented by multifunctional H-cells.

Such a control can be predictable due to the unique properties of microfluidic confinement, which offers ordered transverse diffusion conditions for dissolved macromolecules. Another important aspect of this approach is the use of simple source data for the mathematical model. The initial size distribution of the polymer can be obtained from available analytical tools such as dynamic light scattering data or sedimentation analysis. The resulting microchannel behavior of dissolved species is described by a simple mathematical model that can be processed relatively fast by applicable numerical software. The model offers multiple interrelated factors that control polymer diffusion in H-cells (flow velocity, the ratio of flowrates, and channel length and width). A combination of these factors with numerical software offers a potent tool for designing microfluidic chips with tailored geometry and operation parameters for specific polymer modification needs.

The proposed microfluidic devices can be used as components of lab-on-chip instruments to prepare macromolecular samples for analytical purposes in chemistry and biology, for example, by extracting or cutting-off specific fractions of a macromolecular sample and performing on-chip detection of macromolecules. They can be useful for tuning the molecular size characteristics of polymer samples for further laboratory testing and eliminating undesirable large-size inclusions from polymer solutions with such a microfluidic approach. It should be noted that DLS is a more expensive technique which requires a laboratory-scale instrument and respective set of cuvettes for analysis. A microfluidic setup is a table-scale set of equipment including a typical optical microscope and commercially available pumps for infusion of samples. DLS has limitations of determining sizes of particles to approximately 2–3 nm. With a diffusion model of microfluidic chips, we can apply this technique to analyzing the diffusion behavior of smaller molecules. In addition, the DLS technique is applicable to particles which are close to spherical. Diffusion processing in microfluidic chips can be applied to other shapes of molecules, provided that we modify the mathematical model accordingly. Such a modification will be planned in our future studies.

The proposed method also offers the potential for the development of microfluidic chemical logic devices which can generate a chemical signal represented by the presence of polymer macromolecules at microfluidic chip outlets or their different properties at these outlets. The introduced liquid crystals allow the on-chip detection of such signals in complex lab-on-chip circuits by the automated processing of light transmission in digital images by numerical software and prospective AI tools.

To be critical, in this work we used a 2D model of a 3D microchannel, which is, however, quite a common approach and which provided a good agreement between the numerical and experimental data. In this respect, consideration of 3D microchannel environment in future research may contribute to calculating a more accurate velocity profile in a microchannel and the resulting size distribution of macromolecular samples at outlets. We demonstrated the basic opportunities of polymer detection by liquid crystals in microchannels by analyzing the microchannel environment after polymer processing.

We used a liquid crystal which forms a nematic mesophase that is complementary to the studied polymer. However, thermotropic and lyotropic liquid crystals are characterized by a broad variety of molecular organizations in the mesophase. Therefore, we will apply this liquid crystal approach in the future for a broad range of various systems.

Future research will consider the results of current work and further develop them for more concentrated polymer solutions. The focus of future research will also be the development of the model by introducing more complex diffusion and flow conditions, and considerations for non-diluted solutions will be made. The research will also focus on tuning the optical responses of the liquid crystal matrix in the presence of polymer solutions and polymer–surfactant complexes of various sizes in microchannels and their analysis by future numerical AI tools. In particular, LC microdroplets immobilized on microchannel bottom will be used for the continuous analysis of polymer solutions during H-cell operation.

## 5. Conclusions

In this work, we developed a microfluidic approach for a combined size distribution modification of polymers and their analysis by liquid crystals using polyacrylic acid and MBBA as model substances. PAA processing in H-cells was found to offer a controlled reduction of its intensity peak from about 25 nm to about 10 nm. A Matlab script representing a numerical model for predicting polymer size distribution after microfluidic processing was developed. This model demonstrated a satisfactory agreement with the experimental testing in experiments performed on straight- and serpentine-channel chips with the 15–300 mm lengths and 0.33–1 polymer–solvent flow ratios. The introduction of MBBA liquid crystals with a surfactant as a mediator into an H-cell allowed the visualization of specific microchannel areas occupied by solvent and polymer flows at different flow conditions. These results offer potential applications of H-cell segments as parts of commercial microfluidic chips for combined polymer characterization and microchip analysis and the preparation of polymer samples in laboratory-on-chip devices designed for biomedical analysis.

## Figures and Tables

**Table 1:** Operation parameters of microfluidic chips with the width of the main channel W = 200 µm and respective values of dimensionless numbers.

Channel Length ^1^, mm	Q_0.1_^2^, µL/min	Channel Type	Reynolds Number	Dean Number	Peclet Number × 10^−3^
300	20	Serpentine	1	0.4	60
100	6.7	Serpentine	0.33	0.13	20
30	2	Serpentine	0.1	0.04	6
15	1	Straight	0.05	-	3

^1^ Selected main channel lengths providing diffusion of 10% polymer to Outlet 2 in the flowrate range of 1–20 µL/min. ^2^ Respective flowrates providing diffusion of 10% polymer (with respect to its initial concentration) to Outlet 2 for specific main channel length.

**Table 2:** DLS results of the samples taken at the “Diffused” microchip outlet and their comparison with numerical simulation results.

Experiment	Channel Length, mm	Q_P_ + Q_S_, µL/min	Q_P_/Q_S_	Intensity Peak, Numerical, nm	Intensity Peak, Experiment, nm ^1^	C_P_ at Outlet 2, % of the Initial
1	300	2	1	20	24 ± 4	40
2	300	6	0.33	14	13 ± 2	8
3 ^2^	100	2	0.5	16	18 ± 3	17
4	100	6	0.8	15	18 ± 4	10
5	30	2	0.5	11	9 ± 2	5
6	15	6	0.8	13	12 ± 3	2

^1^ The initial experimental intensity peak is about 31 ± 9 nm. ^2^ Concentration and size distribution curves are shown in Figure 3 and Figure 4.

## Data Availability

Data are contained within the article and supplementary materials.

## Supplementary Materials

The following supporting information can be downloaded at: https://www.mdpi.com/article/10.3390/polym17010028/s1. Figure S1: Microfluidic chip geometry with transverse diffusion of dissolved species to a solvent flow. S—solvent; P0—initial polymer solution; P1 and P2—polymer solutions sampled at the chip polymer and buffer outlets, respectively; x and y—coordinate axes with L and W—main channel length and width, respectively; y_P_—the width of the polymer solution flow after the junction of the inlets.; Figure S2: Correlation between intensity and number size distribution of the initial PAA sample according to DLS data. Reference [21] is cited in the Supplementary Materials.
