# Supplementary material for: Polymer Solutions in Microflows: Tracking and Control over Size Distribution
Source: Polymers (Basel). 2024 Dec 26;17(1):28. doi: 10.3390/polym17010028 (PMC11722862; doi:10.3390/polym17010028)
Supplement: Supplementary file 1 [file polymers-17-00028-s001.zip › polymers-3364509-supplementary.pdf]

**Mathematical Model for Polymer Diffusion in an H-cell Microchip**

The geometry of an H-filter is shown in Fig. S1.

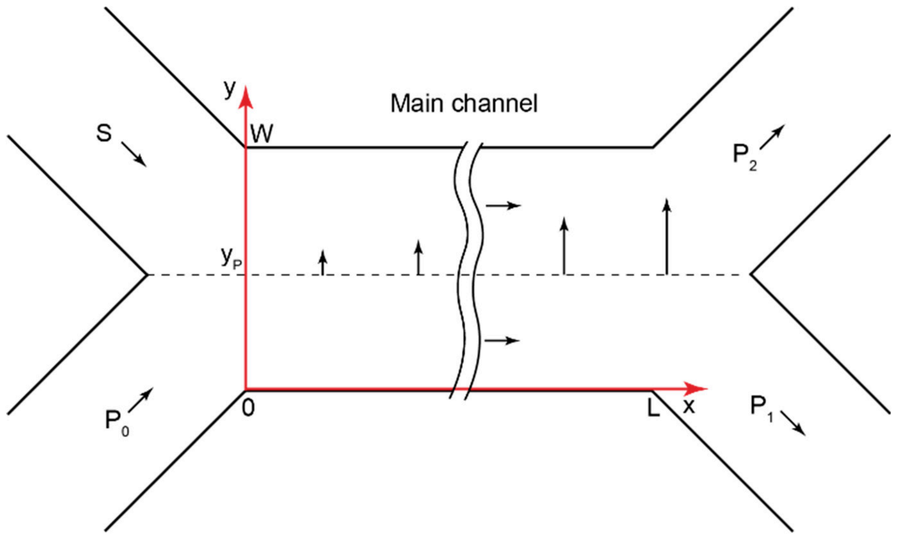


Figure S1. Microfluidic chip geometry with transverse diffusion of dissolved species to a solvent flow. S – solvent; $P_0$ – initial polymer solution; $P_1$ and $P_2$ – polymer solutions sampled at the chip polymer and buffer outlets, respectively; x and y – coordinate axes with L and W – main channel length and width, respectively; $y_P$ – the width of the polymer solution flow after the junction of the inlets.

Let's start with a general convection-diffusion equation [1]:

$$C_{P}{}'_{\tau} + U(y)\nabla C_P = D_P \Delta C_P \tag{S1}$$

where $C_P$ is the molar concentration of PAA with respect to its monomer units, $D_P$ is the diffusion coefficient of the monomer groups (equal to that of polymer macromolecules), U(y) is the flow velocity, ∇ is the Nabla operator Δ is the Laplacian operator, and x is the axial coordinate of a point in a microchannel.

For steady-state flow conditions [1]:

$$U(y)\nabla C_P = D_P \Delta C_P \tag{S2}$$

To simplify this equation, consider only the axial convection in pressure-driven laminar flows in microchannels, the radial diffusion of reacting species and a steady state flow of the reacting species. We also develop the 2D model of microchannel flow, which is a common approach in microfluidic numerical simulations [1,2].

$$U\frac{\partial C_P}{\partial x} = D_{P_i}\frac{\partial^2 C_P}{\partial y^2} \tag{S3}$$

where x and y are axial and radial coordinates in the main channel (Fig. S1).

The Eq. (S3) is a second-order partial differential equation with a constant diffusion coefficient $D_P$.

For a polydisperse polymer, diffusion coefficients of macromolecules depend on their size. In this work, we use the following approach to consider non-constant diffusion coefficient: Eq. S3 can be transformed into a system of equations with various diffusion coefficients $D_{Pi}$. These coefficients can be obtained from a source dynamic light scattering data of the initial polymer sample:

$$\begin{cases} U\frac{\partial C_{P_1}}{\partial x} = D_{P_1}\frac{\partial^2 C_{P_1}}{\partial y^2} \\ U\frac{\partial C_{P_2}}{\partial x} = D_{P_2}\frac{\partial^2 C_{P_2}}{\partial y^2} \\ \dots \\ U\frac{\partial C_{P_i}}{\partial x} = D_{P_i}\frac{\partial^2 C_{P_i}}{\partial y^2} \end{cases} \quad \text{(S4)}$$

where $C_{P_i}$ is the concentration of a PAA in a fraction and $D_{P_i}$ is average diffusion coefficient of macromolecules in this fraction.

The flow velocity U distribution in a microchannel can be approximated by the following equation, which represents a parabolic flow profile:

$$U(y, z) = \beta(y,)U \quad \text{(S5)}$$

where U = Q/(WH) is the flow velocity calculated from the flowrate, W and H are the microchannel's width and height, respectively, and β is the function describing the parabolic flow velocity distribution in a microchannel [1]:

$$\beta = \frac{3}{2}[1 - (2\frac{y}{W} - 1)^2 \quad \text{(S6)}$$

The boundary conditions for the junction of the flows were introduced according to the microchannel geometry shown in Fig. S1:

$$C_P(x = 0, y) = \begin{cases} C^0_{P_i}, y \geq y_P \\ 0, y < y_P \end{cases} \quad \text{(S7)}$$

Assuming zero flow of dissolved species through the walls [1], we get boundary conditions at the walls:

$$\frac{\partial C_{P_i}}{\partial y}_{(0,W)} = 0 \quad \text{(S8)}$$

Eq. S4 can be solved for each fraction according to get the concentration distribution of all the polymer fractions across and along the microchannel.

The concentration of each polymer fraction at the upper $P_2$ Outlet can be calculated as follows:

$$C_{P_i} = \int_{y_P}^{W} C_{P_i}(y_L)\, dy) \quad \text{(S9)}$$

where $C_{P_i}(y_L)$ is the concentration distribution of a polymer fraction across the main channel end (x = L).

The total concentration of polymer diffused to the upper $P_2$ Outlet:

$$C_{P_2} = \sum(\int_{y_P}^{W} C_{P_i}(y_L)\, dy) \quad \text{(S10)}$$

The concentration of each fraction is proportional to the number of macromolecules representing this fraction. Therefore, the ratio:

$$f_i = \frac{C_{P_i}}{C_{P_2}} \quad \text{(S11)}$$

will give the percentage of macromolecules with the diffusion coefficient $D_i$ and the resulting radius $R_i$ (calculated from the Stokes-Einstein equation assuming that the shape of macromolecular coils is close to spherical) in the new size distribution by number of the polymer solution in the $P_2$ Outlet:

$$N(R) = f_i(R_i) \quad \text{(S12)}$$

This mathematical model was implemented into the respective Matlab script.

**Transformation of Numerical Modeling Data from Size Distribution by Number to Size Distribution by Intensity of Scattered Light**

Convection-diffusion equations allow to calculate concentration distribution of particles, such as polymer macromolecules in solution. Concentration is proportional to the number of these particles. Therefore, the source DLS data of the initial PAA size distribution that we used were the size distribution by number.

Size distribution by intensity is, however, a primary DLS report, which is calculated directly from light scattering data. It is more reasonable to compare the size distribution of diffused polymer samples with the initial size distribution of polymer macromolecules by intensity.

The size distribution by number can be calculated from solution of the system of the system of equations (S4).

Dynamic light scattering data of the initial polymer solution were analyzed to identify correlation between its intensity and number size distribution. The DLS software report provides the size distribution both by number and intensity. These data were analyzed to identify correlation between its intensity and number size distribution. We analyzed the ratio of percentages (Intensity/number) vs size.

In the coordinates log(Intensity/Number) vs. log(size) the resulting plot is linear. (Fig. S2). It demonstrates that large particles make a predominant contribution to the intensity of scattered light according to the Rayleigh scattering law (I ~ $R^6$), where I is the intensity of scattered light and R is the radius of a particle.

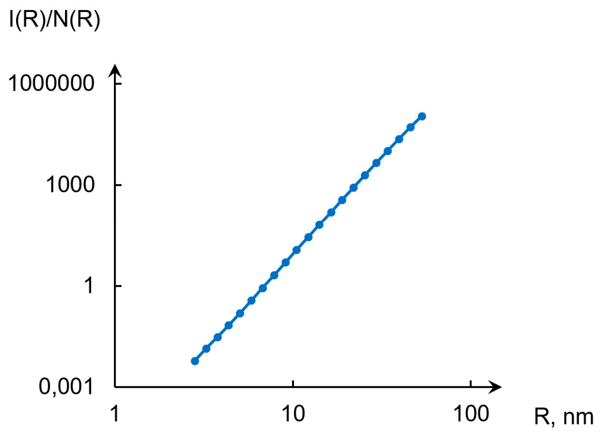


Figure S2. Correlation between intensity and number size distribution of the initial PAA sample according to DLS data.

With this dependence, we can recalculate the size distribution by number data in Matlab back to size distribution by intensity. In the Matlab script, we use correlation coefficients, that were calculated for each point of the plot shown in Fig. S2.

## Scaling Law for Polymer Diffusion

Let's evaluate the time required for polymer macromolecules to diffuse from the polymer flow to the solvent flow (Fig. S1) at the its final point near the P2 Outlet (x = L and y = W).

The time required for such diffusion and the required microchannel length for diffusion to occur is [1]:

$$t_{dif} = \frac{(W - y_P)^2}{4D_P} \tag{S13}$$

where $D_P$ is the diffusion coefficient of polymer macromolecules. For convenience, assume this diffusion coefficient corresponding to the peak at the size distribution by DLS. On the other hand, the time of flow from the junction of the inlets to the end of the main channel is:

$$t_{flow} \approx \frac{L}{3/2U} \tag{S14}$$

where 3/2U is average flow velocity at the central part of the main channel [1]. Therefore:

$$\frac{(W - y_P)^2}{4D_P} \approx \frac{L}{3/2U} \tag{S15}$$

Consider that the width of the polymer flow is proportional to ratio of polymer flowrate and the total flowrate:

$$y_P = W\frac{Q_P}{Q_P+Q_S}, \tag{S16}$$

Then:

$$W - y_P = W - W\frac{Q_P}{Q_P + Q_S} = W(1 - \frac{Q_P}{Q_P + Q_S}) \tag{S17}$$

Continue transformations:

$$W - y_P = W\frac{Q_S}{Q_P + Q_S} = W\frac{1}{\frac{Q_P}{Q_S} + 1} \tag{S18}$$

A convenient dimensionless parameter appears here: the ratio of polymer and solvent flowrates. This parameter will be further used to control size distribution changes of polymer samples in H-cells:

$$Q_N = \frac{Q_P}{Q_S} \tag{S19}$$

Continue transformations:

$$\frac{W^2}{(1 + Q_N)^2 4D_P} \approx \frac{L}{3/2U} \tag{S20}$$

Then:

$$\frac{UW}{D_P} \approx \frac{8}{3}\frac{L}{W}(1 + Q_N)^2 \tag{S21}$$

By introducing a set of dimensionless numbers, we can transform the equation (S21) into the scaling law for the diffusion of polymer macromolecules to the x = L and y = W point. These numbers are Peclet number Pe = UW/$D_P$, the microchannel length-to-width ratio $L_N$ = L/W, and the ratio of polymer and solvent flowrates $Q_N$ = $Q_P$/$Q_S$. The resulting scaling law is:

$$Pe_{dif} \approx \frac{8}{3}L_N(1 + Q_N)^2 \tag{S22}$$

This law includes only dimensionless parameters and considers microchannel geometry and flow conditions. With this law, we can determine factors that are favorable and unfavorable for diffusion to x = L and y = W.

In the less compact form, which is more demonstrative with respect to applicable factors, Eq. S22 looks as follows:

$$\frac{UW^2}{D_P} \approx \frac{8}{3}L(1 + Q_N)^2 \tag{S23}$$

Increase of the channel length L and the flowrate ratio $Q_N$ will increase polymer concentration at x = L an y = W. In turn, increase of the flow velocity U and channel width W will decrease polymer concentration at this point. A combination of these interrelated factors can be used to control concentrations of polymer fractions at the $P_2$ Outlet (Fig. S1) and, therefore, the resulting size distribution of polymer.

## Matlab Script for Polymer Size Distribution

```
%Polymer_Size_Distribution
%First set global variables to be used by all the functions in
this script:
global k p Fraction Size D D0 H L U Q1 Q2;

%INPUT THE VALUES, WHICH CHARACTERIZE THE MICROFLUIDIC SYSTEM
AND POLYMER:

% Input channel diameter D0, µm; channel height, H, µm; and
channel length, L, mm:
D0=200; H=105; L=15;
%Input the flowrate of polymer Q1 and buffer Q2, µl/min:
Q1=2.7; Q2=3.3;

%Calculate the flow velocity U, mm/s:
U=(Q1+Q2)/(D0*H)*10^6/60;

%Enter DLS source data:
%Select  data file name:
%Put the source file in a convenient directory and set the
pathway,
%respectively.
%The file must include three columns: size (1), intensity
percent (2), and
%number percent (3)
T1 = readtable('D:/DLS_Source.xlsx');
%Transform the data into Matlab array:
A = table2array(T1);

%Extract distribution by intensity (I) from the source data:
SizeI = A(:,1);
FractionI = A(:,2);

%Extract distribution by number from the source data:
Size = A(:,1);
Fraction = A(:,3);

%Calculate diffusion coefficients of polymer macromolecules from
the DLS data:
kb = 1.3806488*10^-23;
T = 298;
n = 8.9*10^-4;
Diam = Size;
D = kb*T/(6*pi*n*0.5*10^-9)*10^12./Diam;

%Introduce correlation coefficient for number to intensity
reverse transformation:
Corr = FractionI./Fraction;

%CREATE AND SOLVE SYSTEM OF PARTIAL DIFFERENTIAL EQUATIONS:
%Create the mesh:
```

```
b=1;
a=1;
m = 0;
mesh = 100;
x = linspace(0,a,mesh);
t = linspace(0,b,mesh);

%Solve the PDEs:
sol = pdepe(m,@pdex4pde,@pdex4ic,@pdex4bc,x,t);

%Extract solution (polymer concentrations and their fractions)
at the output of the
%microchannel:
u = sol(end,:,:);
[~,r,c] = size(u);
u = u(:,:);
u = reshape(u,[r,c])';
contrib = Fraction.*u;
tot = sum(contrib);

%Select upper microchannel output for calculating new size
distribution:
bndr = x>=.5;

%Calculate relative concentration of polymer at this output:
h1 = trapz(tot(x>0.5))./numel(tot(x>0.5));

%Calculate new size distribution by number and intensity at this
output:
indcont0 = trapz(sol(end,bndr,:));
indcont = indcont0(:).*Fraction;
aftercontrib = indcont./sum(indcont);
indcontI = indcont(:).*Corr;
aftercontribI = indcontI./sum(indcontI);

%Interpolate size distributio curves:
Size1 = linspace(Size(1),Size(end),500);
aftercontrib1 = interp1(Size,aftercontrib,Size1,'spline');
Fraction1 = interp1(Size,Fraction,Size1,'spline');
FractionI_1 = interp1(Size,FractionI,Size1,'spline');
aftercontribI_1 = interp1(Size,aftercontribI,Size1,'spline');

%Plot original and new size distribution curves:
figure
semilogx(Size1,FractionI_1.*0.01,Size1,aftercontribI_1);
axis([1 500 0 .2])

figure
plot(x,u);


%INTRODUCE PDE COEFFICIENTS AND BOUNDARY CONDITIONS:
%Allocate the variables for PDE functions:
```

```
k = ones(1,length(Size))';
p = zeros(1,length(Size))';
% ---------------------------------------------------------------
function [c,f,s] = pdex4pde(x,~,~,DuDx)
global k D D0 L p U;
pois = 3/2*(1-(2.*x-1).^2);
c = pois.*U*D0*D0/L.*k;
f = D.* DuDx;
s = p;
end
% ---------------------------------------------------------------
function u0 = pdex4ic(x)
global Q1 Q2 k
in = x<=Q1/(Q1+Q2);
u0 = in.*k;
end
% ---------------------------------------------------------------
function [pl,ql,pr,qr] = pdex4bc(~,ul,~,ur,~)
global k;
pl = ul(k);
ql = k;
pr = ur(k);
qr = k;
end
```

References


1. Berthier, J.; Silberzan, P., *Microfluidics for Biotechnology, Second Edition*. Artech House: London, 2009; p 512.
